# Simulation-based homozygosity mapping with the GAW14 COGA dataset on alcoholism

**DOI:** 10.1186/1471-2156-6-S1-S35

**Published:** 2005-12-30

**Authors:** Ondrej Libiger, Nicholas J Schork

**Affiliations:** 1 Polymorphism Research Laboratory, Department of Psychiatry, The University of California, San Diego, La Jolla, California, 92093-0603, USA; 2 Division of Biostatistics, Department of Family and Preventive Medicine, The University of California, San Diego. La Jolla, California, 92093-0603, USA

## Abstract

**Background:**

We have developed a simulation-based approach to the analysis of shared homozygous chromosomal segments and have applied it to data on allele sharing among alcoholics in a single Collaborative Study on the Genetics of Alcoholism pedigree. Our assessment of sharing involved the use of a single-nucleotide polymorphism (SNP) marker map provided by Affymetrix.

**Results:**

All 11 affected individuals in the selected pedigree shared 2 copies of an allele at 4 adjacent SNPs in a region on chromosome 5. Via simulation, we determined that the probability that such sharing is caused by mere chance is less than 0.0000001. After correcting for undocumented inbreeding, this probability rose to 0.0016. The probability that the shared segment emanates from a single ancestor and is unrelated to the affection status is less than 0.0000001 in the corrected pedigree. Haplotype association analysis and a search for a protective locus using unaffected individuals yielded no significant results.

**Conclusion:**

Homozygosity mapping results on chromosome 5 provide suggestive evidence of the region's role as one that may harbor a genetic determinant of alcoholism. Furthermore, the probabilities of chance homozygous allele sharing for the original and for the inbreeding-corrected pedigree provide insight into the impact that inbreeding can have on such calculations.

## Background

Homozygosity mapping has proven to be an effective approach in the identification of chromosomal regions that harbor autosomal recessive genes that influence disease susceptibility [1]. The intuition behind homozygosity mapping is simple: long stretches of genome, for which related individuals affected by a disease all possess 2 copies of the same alleles at many loci, likely reflect the fact that those individuals have inherited 2 copies of a single chromosomal segment from common ancestors that harbors a disease influencing locus. Recently Broman and Weber discussed the potential utility of homozygosity mapping for complex traits but did not discuss the relative advantages of different analysis approaches to the probabilistic assessment of homozygous allele sharing [2].

In order to make compelling claims about the probability that individuals actually share a segment of a chromosome homozygous by descent, two issues must be addressed. First, one must compute the probability that observed sharing of homozygous alleles at adjacent loci actually reflects sharing of a common ancestral segment and not just identity-by-state (IBS) allele sharing of a type that can occur purely by chance. Second, one must determine the expected amount of identity-by-descent (IBD) sharing unrelated to the disease. We have developed and applied a simulation-based approach to the analysis of shared homozygous chromosomal segments and have applied it to data on allele sharing among alcoholics in a single Collaborative Study on the Genetics of Alcoholism (COGA) pedigree.

## Methods

### Pedigree

**Figure 1:**
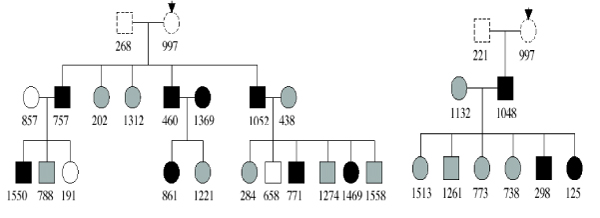
**COGA pedigree 10022. **The 11 individuals with black shading were considered to be affected, the unshaded individuals in the last 2 generations (857, 191, and 658) were considered unaffected, and the gray individuals were treated as unknown. There was no available information about the phenotypes or genotypes of the 3 distinct founders in the first generation (268, 997, and 221). Note: The 2 circles in the first generation that are marked with an arrow represent a single individual.

In order to combat possible allelic and locus heterogeneity, we decided to examine allele sharing among individuals within a single pedigree, COGA pedigree number 10022 (Figure 1).

### Affection status

We considered individuals with a combined score for ALDX1 and ALDX2 of 8 or more as affected. Individuals with score of 2 were considered unaffected.

### Marker map

Simulations described below involving the pedigree suggest that, even after correcting for inbreeding (see Results, Simulations), the expected size of an autozygous segment shared among the affecteds in pedigree 10022 is less than 18.6 Mb (authors' unpublished data). In the provided microsatellite scan, a segment this long would, on average, be marked by only 2 adjacent microsatellites. Actual autozygous regions would then be difficult to distinguish from regions represented by 2 markers shared IBS. For this reason, we decided to use the denser single-nucleotide polymorphism (SNP) map. Specifically, we used the cleaned dataset provided by Affymetrix.

### Simulation studies

We developed a program that simulates chromosome inheritance in a given pedigree and outputs the frequency of shared homozygous loci, autozygous segments, and segments of different lengths shared by a given group of related individuals in that pedigree. Our simulation studies can discern autozygosity from chance homozygosity. We can also infer the conditional probability that a given segment is autozygous when it is observed as homozygous. These simulations are tailored to the precise location, marker density, and marker informativity associated with the observed data. Because recombination rate affects the number and size of homozygous segments (author's unpublished data), we designed the simulation method so that it utilizes empirical recombination rates to model recombination events. In this study, we used empirical recombination rates provided by Affymetrix. The following is a step-by-step description of the simulation procedure:

1) Assign each founder unique chromosomal identification (ID) numbers

2) Use detailed empirical information about chromosome lengths

3) At each locus of relevance, assign each founder marker locus alleles that supervene on the chromosome ID numbers using allele frequency information or strict assignment of particular alleles

4) Simulate recombination to assign chromosomes to each offspring using detailed empirical information about recombination rates

5) Tally IBS/IBD marker allele sharing by examining assigned allele states and underlying chromosomal ID numbers

6) Repeat many times to estimate probabilities of IBS sharing, IBD sharing, and IBD sharing conditional on IBS sharing probabilities based on relative frequencies (note that probabilities will be unique to each locus position due to local recombination rates)

## Results

### Homozygosity mapping in the autosomal genome

For the region on chromosome 5, beginning with locus 49, there appeared to be evidence of significant sharing: all 11 affected individuals shared 2 copies of an allele at 4 adjacent SNPs. Furthermore, 1 of the 2 unaffected individuals did not exhibit this pattern, and was not homozygous in this region. Unfortunately, the genotype of the second unaffected individual was incomplete. Some of the members who are neither affected nor unaffected (the combined scores of ALDX1 and ALDX2 equal to 4 or 6) shared the haplotype and some did not. Individuals 857, 438, and 1132 each carry one copy of the haplotype, which explains the observed homozygosity of their offspring.

### Correcting for inbreeding

**Figure 2:**
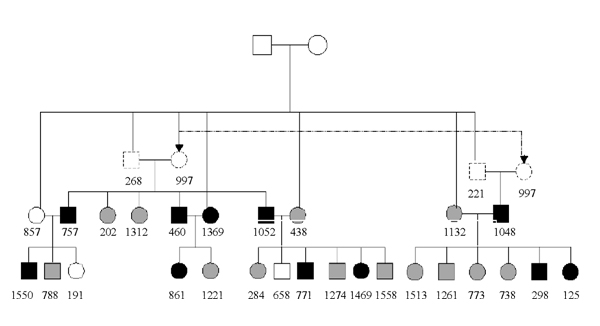
**The inbreeding-corrected pedigree. **The second, third and fourth generations correspond to COGA pedigree 10022 depicted in Figure 1.

One important issue concerns the probability that the shared haplotype comes from a single ancestor. In the case of pedigree 10022, it is impossible that the affected members each share 2 copies of segment emanating from a single ancestor within the pedigree. Either the frequency of the haplotype in the population is high, and it entered the pedigree through several pedigree founders, or inbreeding is present in the generations preceding the founders' generations. We attempted to assess levels of inbreeding among pedigree members by counting the number of homozygous loci in the offspring of the founder pair (268 and 997), and comparing it to simulated counts from arbitrary inbred pedigree structures, specifically, the offspring of siblings, cousins, and second cousins. The average proportion of homozygous loci in 4 offspring in pedigree 10022 (69.7%) was less than in the offspring of sibs (70.7%), but greater than the proportion in offspring of cousins (67.8%). Since unaccounted inbreeding affects the haplotype sharing analysis, all subsequent calculations were also performed for pedigree 10022 with an additional generation consisting of the parents of the original founders (individuals 268, 997, 857, 438, 1369, 221, and 1132), which is depicted in Figure 2. This correction should provide a conservative estimate of the probabilities, because it overestimates the observed amount of inbreeding.

### Simulations

The significance of the observed chromosomal sharing was assessed by simulating the inheritance of marker alleles and chromosome segments in the pedigree. The simulations were performed 10,000,000 times using the provided marker allele frequencies and inter-marker distances. By tracking the number of chromosomal segments in the inbreeding-corrected pedigree, we were able to determine the probability that the shared segment on chromosome 5 emanated from a single ancestor within the pedigree, and is unrelated to the affection status. This probability was less than 0.0000001 (two offspring of siblings share on average 5.8% of autosomal genome (author's unpublished data)). This result suggests that we encountered such an unlikely pattern of sharing due to ascertaining a pedigree with a high density of affected members or that the shared haplotype is common in the population, and entered the pedigree through different ancestors. The fact that the married-ins in the second generation are all carriers of the haplotype supports the latter explanation. Simulation studies were also used to address this question. In analysis of the pedigree without the correction for inbreeding, the probability of observing the sharing pattern found in affected members of pedigree 10022 was less than 0.0000001. However, when we accounted for possible inbreeding, the probability rose to 0.0016.

## Discussion

Our aim in this study was to trace the number and size of shared chromosomal segments emanating from a common ancestor within the pedigree. Therefore, the simulation method assigned founder alleles assuming linkage equilibrium. The presence of strong linkage disequilibrium would modify the relative frequencies of sharing. However, since this pedigree was ascertained from an outbred population, we assumed that the effect of linkage disequilibrium on the relative frequencies would be negligible compared to the effect caused by inbreeding.

Our results suggest that members of the investigated pedigree exhibit chromosomal sharing patterns compatible with a pedigree in which all founders are siblings. The patterns of sharing are also possible with different pedigree structures that would influence chromosome segment sharing probabilities. More sophisticated extraction of information on the pattern of inbreeding would make the calculations more reliable. A method for accurately estimating the inbreeding coefficient has been recently proposed by Leutenegger et al. [3]. However, as Clark noted, the inbreeding coefficient does not characterize sufficiently the size distribution of autozygous segments, because one can have the same inbreeding coefficient with different paths of common ancestry [4]. Therefore, a method estimating paths of co-ancestry, which would exploit the observed size distribution of shared chromosomal segments, would be beneficial.

The low probabilities of IBS chromosomal sharing, as well as IBD chromosomal sharing of the segment containing the proposed "disease" locus suggest that the haplotype is relatively common in the population from which the pedigree is sampled. The high frequency of the haplotype among the affected individuals may not be due to the fact that this region is associated with alcoholism. It might instead harbor a locus influencing a different trait common to the family under study. Analysis of unaffected individuals could provide stronger evidence for the role of this chromosomal region in the genetic predisposition of alcoholism. Unfortunately, this pedigree does not contain many unaffected individuals whose haplotypes could be analyzed. The additional haplotype association analysis and the search for a protective allele yielded no significant results.

## Conclusion

The results of our analyses suggest that the genetic basis of alcoholism, even when considered in context of a single pedigree, is still very complex. Our analysis of shared homozygous segments in the original and in the inbreeding-corrected COGA pedigree suggests that there is a region of shared homozygosity that isn't likely to occur by chance. This segment may harbor an alcoholism susceptibility locus. Further, the observed sharing has low probability even when the pedigree is corrected for inbreeding.

## Abbreviations

COGA: Collaborative Study on the Genetics of Alcoholism

IBD: Identity by descent

IBS: Identity by state

ID: Identification

SNP: Single-nucleotide polymorphism

## Authors' contributions

OL implemented the simulations method, carried out the analyses, and drafted the manuscript. NJS invented the simulation method and revised the manuscript. Both authors read and approved the final manuscript.
